# A comparison of the effects of sheep's milk and cow's milk on recovery from eccentric exercise

**DOI:** 10.3389/fspor.2023.1335434

**Published:** 2024-01-11

**Authors:** Ben Ravenwood, Jane Coad, Matthew J. Barnes

**Affiliations:** ^1^ School of Sport, Exercise & Nutrition, Massey University, Palmerston North, New Zealand; ^2^ School of Food and Advanced Technology, Massey University, Palmerston North, New Zealand

**Keywords:** sheep’s milk, cow’s milk, eccentric exercise, muscle damage, recovery, soreness

## Abstract

**Introduction:**

When consumed after eccentric exercise, cow's milk has been shown to improve recovery and alleviate symptoms of exercise induced muscle damage. Although currently less commercially available than cow's milk, sheep's milk may offer similar or greater benefits for recovery as it is higher in protein and energy; however, the effect of sheep's milk in any exercise context has not been explored. This study compared the effects of a sheep's milk beverage and a cow's milk beverage on recovery from strenuous eccentric exercise. Additionally, the effects of each beverage on satiety and gastrointestinal comfort were assessed.

**Methods:**

Ten healthy males completed baseline measures of perceived muscle soreness and maximal voluntary concentric, eccentric, and isometric quadriceps force of one leg before completing 200 maximal eccentric knee extensions on an isokinetic dynamometer. Measures were repeated 0.5, 24, 48 and 72 h post-eccentric exercise. After 0.5 h measures, participants consumed either 450 ml of chocolate flavored sheep's milk or chocolate flavored cow's milk. Following a washout period, participants completed a second trial on the contralateral leg and consumed the other beverage. Additionally, a satiety and gastrointestinal comfort questionnaire was completed before and after each beverage was consumed.

**Results:**

Eccentric exercise brought about a significant decrease in muscle function over time (all *P* < 0.012). No difference between treatments (all *P* > 0.097) was found. Measures of muscle soreness increased over time (all *P* < 0.002), however no difference was observed between treatments (all *P* > 0.072). Only sheep's milk altered perceived satiety, however, only the response to “How full do you feel” differed between treatments (*P* = 0.04).

**Discussion:**

The results of this study suggest that consuming sheep's milk may provide similar benefits as cow's milk when recovering from exercise-induced muscle damage. While these findings provide initial support for the use of sheep's milk in a muscle recovery context, further research is warranted to confirm these findings. Given its superior nutritional profile, greater impact on satiety and lower environment impact, sheep's milk may be a more efficient post-exercise recovery beverage, compared to cow's milk.

## 1 Introduction

The sports industry is constantly investigating new technologies and ergogenic aids so that athletes can perform at their peak for as long as possible. Recovery from exercise-induced muscle damage (EIMD) has been identified as an important factor in maintaining performance, as it can impair muscular strength and power (1), which in turn affects athletic performance (2). As post-exercise nutrition can play a significant role in how well an athlete recovers after exercise (3), the effects of various nutritional interventions on recovery from EIMD have been investigated (4, 5).

When consumed after strenuous, damaging exercise, cow's milk and cow's milk-based protein-carbohydrate beverages have been shown to positively alter the symptoms of EIMD, when compared to carbohydrate or water. Consuming cow's milk after damaging exercise has been shown to enhance performance recovery, including measures of force (6–9), speed (9–11), agility, team sport related running (10) and counter movement jump height (9, 11). Additionally, drinking cow's milk can result in decreased levels of creatine kinase (6, 9), myoglobin (6) and reduced delayed onset of muscle soreness (9) in the days after exercise. Together, these results suggest that cow's milk is a viable option for enhancing recovery from EIMD (12).

While currently less commercially available than cow's milk, sheep's milk may offer a nutritionally superior and more environmentally friendly option for athletes wanting to expedite recovery from EIMD. Sheep's milk has a higher percentage of milk solids, including ∼60% more protein and fat, 60% more leucine, an amino acid associated with the stimulation of protein synthesis (13), and slightly higher amounts of carbohydrates, when compared to cow's milk (14). Despite its greater protein content and energy density, sheep's milk production is deemed to be more environmentally friendly than the production of cow's milk (15, 16); this may be an important consideration as an increasing number of consumers favor foods that are produced with a lower environmental impact (17).

Classified as an A2 milk, because it contains only the A2-β casein and no A1-β casein (18), sheep's milk may be an option for those who suffer from A1 milk intolerance or allergies (19); this may impact up to 17% of the population (20). When consumed after muscle damaging exercise, A2 milk appears to provide similar recovery benefits to A1 milk, leading Kirk et al. (11) to suggest that A2 milk may be a useful ergogenic aid for athletes who have an intolerance to A1 milk. Although A2 cow's milk was used by Kirk et al. (11), a similar effect may be expected when consuming sheep's milk, particularly given its higher protein and energy content. However, to date, there appears to be no research on the use of sheep's milk in a sporting context, particularly in comparison to cow's milk. Therefore, the aim of this study was to compare the effects of sheep's milk and cow's milk on recovery from strenuous eccentric exercise. Additionally, the effects of each beverage on satiety and gastrointestinal comfort were assessed.

## 2 Materials and methods

### 2.1 Study design

A randomized, double-blind, cross-over design was used to compare the effects of sheep's milk and cow's milk on recovery from EIMD. Ten healthy men volunteered to participate. Briefly, at baseline, muscle soreness and maximal voluntary concentric, eccentric and isometric quadriceps force, of one leg, was assessed. Participants then performed 200 maximal eccentric contractions to induce muscle damage. Thirty minutes after the muscle damage protocol, muscle soreness and muscle function tests were performed again, before participants consumed either a chocolate flavored sheep's milk beverage or a chocolate flavored cow's milk beverage. Participants returned to the laboratory 24 h, 48 h, and 72 h later for follow up measures of soreness and muscle function. Following a washout period of at least 14 days, participants completed the same protocol using the other leg and consumed the other milk beverage. Beverage and leg used for each trial were randomly allocated in a counter-balanced fashion, using a single cross-over design.

### 2.2 Participants

Ten healthy, physically active men (age = 24.9 ± 4.3 years, body mass = 82.8 ± 10.3 kg, height = 179.1 ± 6.7 cm) volunteered to take part in this study. All participants had at least two years of resistance training experience at a recreational level (minimum of two trainings per week) and had maintained their normal exercise routine in the two months prior to taking part in the study. For sample size estimation, power analysis was carried out in G*Power (version 3.1.9.7) (21). Based on the effect of cow's milk on recovery of muscle function after damaging exercise (8) (*d *= 0.37), and *α* = 0.05 and power = 0.80, a sample size of *n* = 10 was required. The study protocol was approved by the Massey University Southern A Human Ethics Committee (19/22). Participants completed a pre-exercise health screening questionnaire, to confirm their suitability for participation, and provided written informed consent prior to familiarization. At least seven days before the first experimental trial, participants were familiarized with the measures and exercise protocol used in the study.

For both trials, participants were required to refrain from any exercise (except for any necessary walking), nutritional supplement use, and alcohol consumption in the 48 h before day one of their trial, and for the duration of the trial period itself (72 h). Participants were instructed to record their food intake from 48 h prior to day one of their trial until the end of the trial, using the free “Easy Diet Diary” application on their smart phones, or by recording their food intake on a paper version of the diary. Participants were also asked to replicate their diets from Trial 1 for Trial 2 as closely as possible. Food records for both trials were analyzed using dietary analysis software [FoodWorks 10^©^ Xyris Software (Australia) Pty Ltd.]. Finally, participants were instructed to arrive at the laboratory in the morning, after an overnight fast of at least 10 h, on all data collection days.

### 2.3 Muscle soreness

On arrival at the laboratory, participants were asked to complete a series of subjective muscle soreness measures including stepping-up and down on a 45 cm box using the test leg, and a two-legged squat to 90° knee flexion. Each test was performed twice before the participant reported how sore their leg felt on a visual analogue scale between 0 and 10 (0 = no soreness, 10 = extremely sore) (22). Additionally, a pressure pain threshold test (PPT) was used to assess localized, pressure induced pain, as described by Barnes et al. (23). The highest value of the three measures was used for analysis.

All three tests were carried out at baseline, and at 0.5 h, 24 h, 48 h and 72 h post muscle damage protocol. In cases where visual bruising arose from PPT, the testing sight was adjusted slightly on the belly of the vastus lateralis muscle to avoid potential sensations of pain that were unrelated to the muscle damage protocol.

### 2.4 Muscle function

Following the perceived muscle soreness tests, participants warmed-up on a cycle ergometer (Monark, Varberg, Sweden) for 5 min at 100 W. They were then seated on the isokinetic dynamometer (Biodex Medical Systems, New York, USA) and straps were fitted across the chest, hips and test leg. The ankle of the test leg was strapped to the end of the lever arm of the knee attachment. Range of motion was set from a comfortably flexed knee position (0° start point) and extended to 60° (end point). This range of motion, and seat position settings, were recorded and used in all subsequent performance measures, and the muscle damage protocol. Participants then completed three consecutive repetitions of maximal concentric knee extension, followed by a 2-min passive recovery period, and then three consecutive repetitions of maximal eccentric knee extension. Concentric and eccentric torque was measured at an angular velocity of 30° s^−1^. After a further 2-min passive recovery, quadriceps isometric tension was measured at 75° knee extension by completing three repetitions of 3-s maximal efforts, with each repetition separated by a 10 s rest. Peak torque/tension was recorded for each test. Each performance test was repeated at 0.5 h, 24 h, 48 h and 72 h post muscle damage protocol.

### 2.5 Eccentric exercise protocol

Following baseline muscular performance tests, participants remained on the dynamometer and performed two sets of 100 maximal eccentric contractions using the quadriceps muscle group of the test leg. Each repetition was performed over a 60° range of motion, and at an angular velocity of 30° s^−1^. A 5-min passive recovery period was given between the two sets. Participants received verbal encouragement to resist the downward movement of the dynamometer arm to ensure a constant maximal effort was given throughout the protocol. Similar isokinetic protocols, of various volumes, have been used to investigate the effects of milk-based beverages (6–9), and other nutritional interventions (24, 25). There was no significant difference between the sheep's milk and cow's milk trials for total work completed during the muscle damage protocol (24.2 ± 6.5 kJ and 25.0 ± 5.1 kJ, respectively; *P* = 0.52).

### 2.6 Treatment

Following the 0.5 h muscle soreness and function measures, participants were asked to consume either a chocolate flavored sheep's milk beverage or the equivalent volume of a chocolate flavored cow's milk beverage (450 ml). Based on the previously stated effects of cow's milk on recovery (6–10), the cow's milk beverage was used as a positive control. Additionally, the milk beverages were consumed once, soon after exercise, as this has been shown to be beneficial for recovery after damaging exercise (7–10). To minimize the potential for bias, the treatment was double blinded. Both milks were flavored in the same way, including the addition of 1.5% sucrose per volume. The beverages were provided to participants in clear plastic bottles labelled either “1” or “2”. In an attempt to naturally offset the difference in total energy content between the sheep's milk (whole sheep milk, Fernglen Farm, Masterton, New Zealand) beverage and the cow's milk beverage, full fat cow's milk was used (full cream milk, Anchor, Takanini, Auckland, New Zealand). Proximate analysis was carried out independently by the Nutrition Laboratory of the School of Food and Advanced Technology, Massey University. Table 1 provides a summary of the energy and macronutrient content of both chocolate milk beverages following proximate analysis, and the amino acid concentration of both drinks is provided in Table 2.

**Table 1:** Proximate analysis of the chocolate sheep’s milk and cow’s milk beverages.

Milk component	Sheep's milk			Cow's milk		
	Per 450 ml beverage	% of milk (per 100 ml)	% of milk solids	Per 450 ml beverage	% of milk (per 100 ml)	% of milk solids
Total milk solids (g)	86.5	19.2	100	66.3	14.7	100
Energy (kJ)	1,828.2	(406.3)	-	1,327.3	(295.0)	-
Protein (g)	26.7	5.9	30.9	15.9	3.5	24.0
Fat (g)	23.7	5.3	27.4	15.4	3.4	23.2
Carbohydrate (g)	27.1	6.0	31.4	26.4	5.9	39.8
Lactose (g)	20.3	4.5	23.4	19.8	4.4	29.9
Dietary fibre (g)	4.41	1.0	5.1	4.95	1.1	7.5
Ash (g)	4.6	1.0	5.3	3.7	0.8	5.6
Water (ml)	363.6	80.8	0.0	383.9	85.3	0.0

**Table 2:** Amino acid profile of the chocolate sheep's milk and cow's milk beverages.

Amino acids	Sheep's milk (mg/100 mg)	Sheep's milk (g/450 ml beverage)^a^	Cow's milk (mg/100 mg)	Cow's milk (g/450 ml beverage)^a^
Aspartic acid	0.44	2.05	0.26	1.20
Threonine	0.26	1.21	0.16	0.74
Serine	0.29	1.35	0.17	0.78
Glutamic acid	1.05	4.89	0.64	2.95
Proline	0.57	2.65	0.31	1.43
Glycine	0.11	0.51	0.07	0.32
Alanine	0.21	0.98	0.11	0.51
Valine	0.38	1.77	0.22	1.01
Methionine	0.16	0.74	0.09	0.41
Isoleucine	0.29	1.35	0.17	0.78
Leucine	0.56	2.61	0.33	1.52
Tyrosine	0.28	1.30	0.17	0.78
Phenylalanine	0.27	1.26	0.16	0.74
Histidine	0.15	0.70	0.09	0.41
Lysine	0.49	2.28	0.29	1.34
Arginine	0.19	0.88	0.13	0.60

Amino acid profile tests (acid stable) were carried out independently by the Nutrition Laboratory at the School of Food and Advanced Technology, Massey University.

^a^ Values were calculated using density conversion factors for each milk using the lower value of the ranges documented by Park et al. (2007).

### 2.7 Gastrointestinal comfort and satiety

Upon arrival to the lab on day one of each trial, participants were asked to complete a baseline gastrointestinal comfort (26) and satiety questionnaire (27) before beginning the muscle soreness and function testing. Following the consumption of the allocated milk beverage, participants were asked to remain in the lab for 20 min before completing the gastrointestinal comfort and satiety questionnaire again.

### 2.8 Statistical analysis

Data were analyzed using the Statistical Package for the Social Sciences software (SPSS version 25.0, IBM, New York, USA). Prior to analysis, the Shapiro-Wilk test was used to assess data for normal distribution. A general linear model, two-way, repeated-measures ANOVA (treatment × time) was used to compare treatment conditions over time for subjective muscle soreness, muscle function and gastrointestinal comfort and satiety measures. For all analyses, the Greenhouse–Geisser or Huynh-Feldt correction for the violation of sphericity was applied, where appropriate. *Post hoc* analysis, with Bonferroni adjustment, was made to investigate any significant main or interaction effect. A paired *t*-test was used to compare the total work done during the muscle damage protocol between the sheep's milk and cow's milk trials. Paired *t*-tests were also used to compare dietary intakes (energy, protein, fat and carbohydrate) between treatment groups for both the duration of the trial (five days) and for day one of each trial. Results are reported as means ± standard deviation, and statistical significance was set at *P *< 0.05.

## 3 Results

### 3.1 Sheep's and cow's milk beverage composition

Table 1 compares the composition of the chocolate flavored sheep's milk and chocolate flavored cow's milk beverages, following proximate analysis. The sheep's milk beverage had a higher proportion of total milk solids which included higher amounts of protein and fat, but similar amounts of carbohydrates, compared to the cow's milk beverage. Protein and fat components contributed a higher percentage to total milk solids in the sheep's milk beverage compared to the cow's milk beverage (30.9% protein, 27.4% fat, and 24.0% protein, 23.2% fat, respectively). As such, the carbohydrate contribution, including lactose, to total milk solids was lower in the sheep's milk beverage (sheep's milk: 31.4% carbohydrate and 23.4% lactose; cow's milk: 39.8% carbohydrate and 29.9% lactose).

Participants received 1,828.2 kJ of energy, 26.7 g of protein, 23.7 g of fat, 27.1 g of carbohydrate, or 1,327.3 kJ of energy, 15.9 g of protein, 15.4 g of fat, 26.4 g of carbohydrate, per 450 ml sheep's milk and cow's milk treatment, respectively.

Table 2 compares the amino acid profile of both chocolate milk beverages, as mg per 100 mg milk and as total quantity, as g per 450 ml treatment. The greater protein content in sheep's milk provided higher amounts of all the measured amino acids, compared to the cow's milk beverage. Notably, the branch chain amino acids, leucine, isoleucine and valine were present in much higher amounts in the sheep's milk beverage.

### 3.2 Muscle function

**Figure 1:**
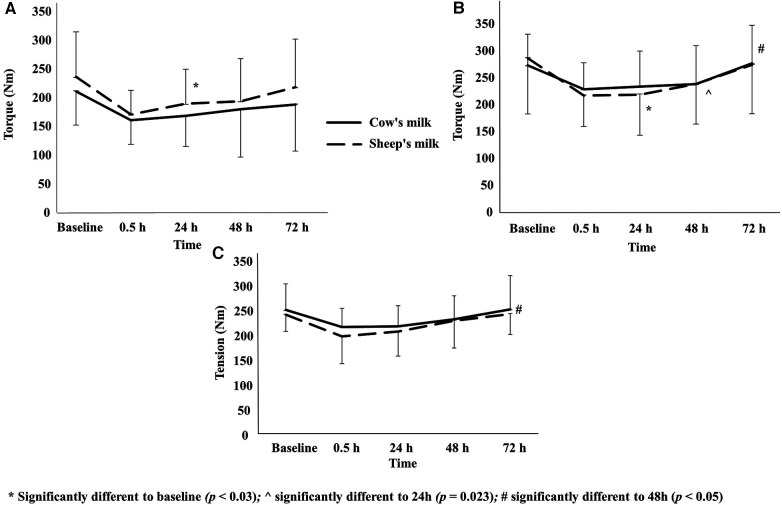
Concentric (**A**), eccentric (**B**) and isometric (**C**) quadriceps force before and after 200 maximal eccentric contractions. Participants consumed either a chocolate sheep’s milk or cow’s milk beverage after exercise (*n* = 10: mean ± SD).

Completion of the muscle damage protocol resulted in significant decreases in peak concentric (*P* = 0.012) and eccentric (*P* = 0.004) torque and isometric tension (*P* = 0.011) over time. No significant treatment effects (all *P *> 0.097) or treatment × time interactions (all *P* > 0.318) were observed for any of the performance measures (Figure 1).

After 24 h, significant decrements in both concentric and eccentric measures were observed in the sheep's milk trial (*P* < 0.05), but not in the cow's milk trial. At 48 h, eccentric torque was significantly greater than at 24 h (*P *= 0.023) in the sheep's milk trial, and at 72 h eccentric torque was significantly greater than 24 h values (*P* = 0.025) in the cow's milk trial. Similarly, there was an improvement in isometric tension at 72 h compared to 24 h values in the cow's milk trial (*P* < 0.033). No other significant differences were observed between baseline values and any of the subsequent measures in both trials.

### 3.3 Muscle soreness

There was a significant time effect across all three measures of muscle soreness/pain (all *P* < 0.002; Table 3), but no significant effects for treatment or treatment × time interactions (all *P* > 0.072). Significant increases in perceived muscle soreness were observed at 0.5 h and 24 h in both trials, for both the step-up and squat measures (*P* < 0.05).

**Table 3:** Perceived muscle soreness (*n* = 10: mean ± SD) recorded during a step up, squat and pressure pain threshold test (PPT) before and after 200 eccentric contractions of the quadriceps. Participants consumed either a sheep's milk beverage (SM) or a cow's milk beverage (CM).

	Baseline	0.5 h	24 h	48 h	72 h
Step-up					
SM	1.7 ± 0.5	4.3 ± 3.0*	3.2 ± 2.2*	2.5 ± 2.3	1.6 ± 2.6
CM	1.6 ± 0.5	5.6 ± 2.7*	4.1 ± 2.8*	3.0 ± 2.9	1.9 ± 3.0
Squat					
SM	1.6 ± 0.5	3.7 ± 2.9*	2.9 ± 1.7*	2.4 ± 2.4	1.6 ± 2.8
CM	1.8 ± 0.7	4.4 ± 3.1*	3.6 ± 3.0*	2.6 ± 3.1	1.7 ± 3.0
PPT (*N*)					
SM	72.0 ± 14.3	70.2 ± 19.5	53.2 ± 12.2*^,^^	55.0 ± 14.3	62.6 ± 13.1
CM	68.0 ± 19.7	69.2 ± 21.7	58.5 ± 14.7*^,^^^,#^	61.0 ± 15.3^#^	65.1 ± 16.3

* Significantly different to baseline (*P* < 0.05). ^Significantly different to 0.5 h (*P* < 0.05). ^#^Significantly different to 72 h (*P* < 0.05).

PPT was significantly different from baseline at 24 h, in both trials (*P* < 0.05). Additionally, significant differences between 0.5 h and 24 h measures were observed in both trials (*P *< 0.04). In the sheep's milk trial, PPT at 72 h was significantly lower than 24 and 48 h scores (*P* < 0.05 and *p* < 0.01, respectively).

### 3.4 Gastrointestinal discomfort and satiety

The satiety questionnaire showed significant time (*P *= 0.005) and treatment×time interactions (*P* = 0.048) for the “How full do you feel?” question, suggesting a difference in satiety effects between the two milk beverages. There were, however, no further differences detected for treatment, time, or treatment × time interactions for all the other questions (all *P* > 0.107).

**Figure 2:**
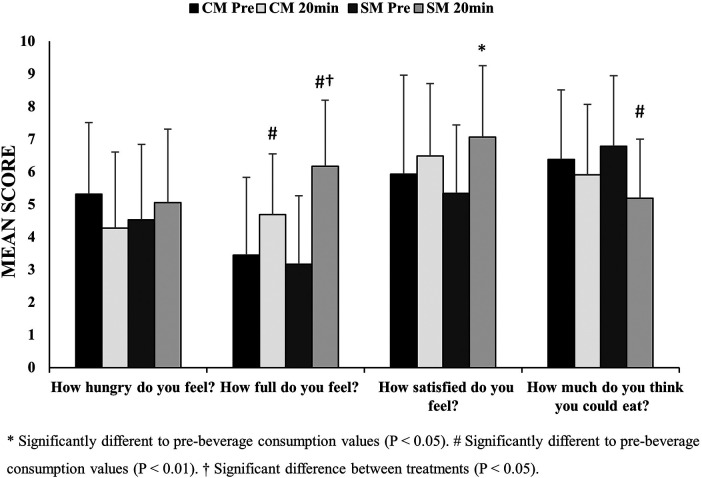
Measures of perceived satiety before (Pre) and 20-min (20 min) after consuming either a chocolate sheep’s milk (SM) or cow’s milk (CM) beverage (*n* = 10: mean ± SD).

Significant increases in feelings of satiety in the sheep's milk trial for three of the four measures (all *P* < 0.05) were reported (Figure 2). No changes were detected in the cow's milk trial (all *P* > 0.05). Additionally, the consumption of the sheep's milk beverage was shown to provide greater feelings of fullness compared to the cow's milk beverage when participants were asked “How full do you feel?” (*P* < 0.05).

Participants also completed a gastrointestinal comfort questionnaire at baseline and 20 min following the consumption of the allocated milk beverage. Mild feelings of discomfort were felt by two participants in the sheep's milk trial and a single participant in the cow's milk trial; all other participants were absent of any gastrointestinal discomfort.

### 3.5 Dietary analysis

A comparison of the total average energy and macronutrient intake for the duration of each trial is displayed in Table 4. Participants recorded their food intake from 48 h before the first trial day until the completion of the trial (five days total). Data from two participants were excluded from the analysis due to having incomplete diaries; food entries were missing for entire days and for entire meals in both trials.

**Table 4:** Energy and macronutrient intakes, and between treatment paired sample *t*-test results, for trial total (sheep's milk (SM) and cow's milk (CM)) and day one of each trial (*n* = 8: mean ± SD).

	Treatment	Mean ± SD	*P* value
Trial total (5 days)			
Energy (kJ)	SM	40,939.23 ± 6,700.66	0.417
	CM	39,041.54 ± 4,464.12	
Protein (g)	SM	539.08 ± 108.82	0.078
	CM	451.03 ± 55.88	
Fat (g)	SM	379.20 ± 61.35	0.476
	CM	356.15 ± 92.86	
Carbohydrate (g)	SM	977.09 ± 307.49	0.738
	CM	1,009.05 ± 118.03	
Day 1 total			
Energy (kJ)	SM	10,390.95 ± 1,756.89	0.520
	CM	9,843.87 ± 1,530.83	
Protein (g)	SM	146.08 ± 34.64	0.330
	CM	129.55 ± 32.71	
Fat (g)	SM	104.14 ± 21.19	0.491
	CM	95.05 ± 28.06	
Carbohydrate (g)	SM	225.79 ± 73.31	0.962
	CM	227.29 ± 38.09	

Total food intake was similar between both trials with no significant differences for energy, fat and carbohydrate intake (all *P* > 0.417). Daily protein intake appeared to be higher in the sheep's milk trial, however differences were not significant (*P* = 0.078). Similarly, there were no significant differences between energy and macronutrient intakes between trials when comparing intakes from the trial days alone (all *P* > 0.330).

## 4 Discussion

Cow's milk has been shown to benefit recovery from EIMD (6–10), however the effects of sheep's milk has not previously been investigated. The current study found that EIMD related changes in force and muscle soreness are similar when a chocolate flavored sheep's milk or cow's milk beverage is consumed after eccentric exercise, despite the sheep's milk beverage containing higher amounts of protein, EEAs, and energy. The higher amounts of milk solids, in particular protein in sheep's milk likely explains the greater feelings of fullness reported after consuming this beverage, whereas consuming cow's milk did not alter feelings of satiety. As a smaller volume of sheep's milk needs to be consumed in order obtain the equivalent amount of nutrition, from cow's milk, that has been shown to benefit recovery, sheep's milk may provide a more efficient, satiating option for those looking to aid recovery from EIMD. Additionally, as sheep's milk is classified as A2, this beverage may be a viable alternative for those who have an intolerance to A1 milk types.

In the current study, whole milk was used from both animal species, and both were flavored in the same way (1.5% added sucrose and natural cocoa flavoring). Similar to previous summaries (28, 29), the independent proximate analysis performed for this study showed that sheep's milk had higher total milk solids, protein and fat content, reasonably higher ash content, and almost identical carbohydrate and lactose content. The higher protein content resulted in 171% more essential amino acids in the sheep's milk beverage (11.92 g vs. 6.95 g). Although the mechanism for cow's milks benefits on recovery is yet to be categorically confirmed (12), it has been suggested that consuming cow's milk may maximally stimulate muscle protein synthesis (MPS) and minimize protein breakdown (8, 10, 11). As this is achieved by consuming 8–10 g of essential amino acids (30), it is likely that the sheep's milk beverage provided an appropriate stimulus for maximal MPS to occur in the post-exercise period.

Voluntary force production significantly decreased, and perceived soreness increased, following the completion of 200 maximal eccentric contractions of the quadriceps. Comparable responses have been reported previously after similar or slightly higher volumes of the same eccentric exercise (25, 31, 32). Although the timeline of recovery differed somewhat between contraction types with each beverage, the absence of treatment or interaction effects suggest that the overall response and recovery was the same for each beverage. The greatest decreases in force, and highest perceived ratings of soreness, were observed 30 min and 24 h following the muscle damage protocol, before returning towards baseline levels by 72 h post-exercise. The lack of difference between beverages may indicate one of two things: that neither beverage improved recovery, or that both improved recovery equally. The use of cow's milk as our control was based on the body of evidence that suggests that cow's milk or cow's milk-based beverages are useful nutritional supplements for athletes recovering from EIMD (6–10). It is worth noting that our cow's milk beverage provided just 1 g less protein, and similar amounts of carbohydrate, than the milk used by others (6, 8–11) and therefore it may be expected to provide similar benefits. However, while Cockburn and colleagues have consistently illustrated that cow's milk is beneficial for improving measures of EIMD, these effects may be modest, ranging from unclear to likely beneficial (6–10), and therefore we cannot simply assume that either treatment has had a positive effect on recovery.

Interestingly, the sheep's milk beverage (26.7 g protein) provided considerably more protein than the cow's milk beverage (15.9 g), and yet no differences in measures of muscle function or soreness were observed between treatments. This lack of difference is in line with the findings of Cockburn et al. (8) who found that a greater volume of milk (500 ml vs. 1,000 ml), and therefore protein (17.4 g vs. 34.8 g protein), did not provide additional benefits for recovery. This, they suggest, is because ∼20 g of protein is likely to optimize MPS, and protein intakes above this amount do not have an added benefit (33). However, whether MPS is responsible for accelerating recovery from EIMD has recently been questioned. Despite showing that symptoms of EIMD can be improved by consuming a supplement containing 20 g of protein and polyphenols, Pavis et al. (31) found that this effect was not related to increases in MPS. Instead, the authors suggest that MPS may be maximal during the first 72 h post-eccentric exercise, irrespective of exogenous protein intake, and that dietary protein, in particular whey, may influence leukocyte numbers and activity, which accelerates muscle recovery. Based on the findings of Pavis et al. (31) more research is needed to understand the mechanisms behind the benefits milk has on recovery from EIMD.

Subjective measures of satiety suggest that sheep's milk had a more satiating effect compared to the cow's milk. Although only responses to the question “How full do you feel?” differed between beverages, all other measures related to satiety changed from baseline after consuming the sheep's milk beverage; cow's milk had no effect on satiety. Considering the differences in macronutrient composition between the two beverages, increased satiation with sheep's milk may be unsurprising, particularly as foods with higher protein content can have greater satiating effects compared to foods containing less protein, even when matched for energy (34).

When considering gastrointestinal comfort, two participants reported feelings of mild discomfort after consuming the sheep's milk beverage, compared to one participant with cow's milk. That there was no significant difference between treatments for gastrointestinal comfort was not unexpected, as participants with known dairy or lactose intolerance were excluded from participating in this study. Although Shrestha et al. (35) reported no differences in gastrointestinal comfort in “dairy avoiding” women who consumed an equal volume of sheep's milk or cow's milk, more research is needed to assess whether sheep's milk is a better option for those suffering from cow's milk related intolerances and allergies.

The major limitation of this study was the absence of a control treatment. As previously discussed, there is evidence to support the effects of cow's milk on recovery from EIMD, however without a negative control it is unclear whether either sheep's or cow's milk improved recovery and minimized soreness in the present study. Although this approach, not to use a placebo, has been used by others to compare the effects of protein supplementation on recovery from EIMD (36), in order to address this limitation, future research should utilize a three armed protocol (8) that includes both a negative (placebo) and positive (cow's milk) control, along with sheep's milk. Additionally, as sheep's milk is energy and protein dense, it is possible that a sheep's milk drink may be a more efficient recovery aid, as a lower volume (∼340 ml) of sheep's milk can provide the same nutrition as volumes of cow's milk that are known to improve recovery (12). Therefore, matching energy and/or protein between beverages may be appropriate in future research.

In conclusion, this study was the first to compare the effects of sheep's milk and cow's milk on recovery from EIMD. Here we have shown that the two milks have the same effect on recovery from strenuous eccentric exercise and, therefore, based on previous research, we suggest that sheep's milk is a viable option for those wanting to improve recovery from EIMD.

## Data Availability

The original contributions presented in the study are included in the article/Supplementary Material, further inquiries can be directed to the corresponding author.
